# Extraovarian fibrothecomas: Two case reports and comprehensive review of ovarian sex cord-stromal fibroma-thecoma tumors

**DOI:** 10.17305/bb.2025.12816

**Published:** 2025-08-18

**Authors:** Nejra Selak, Ivana Čerkez, Ermina Iljazović, Azra Sadiković, Maja Konrad Čustović, Jasminka Mustedanagić Mujanović, Edina Ahmetović Karić

**Affiliations:** 1 Department of Pathology, University Clinical Center Tuzla, Tuzla, Bosnia and Herzegovina; 2 Department of Gynecology, University Clinical Center Tuzla, Tuzla, Bosnia and Herzegovina

**Keywords:** Fibrothecoma, extraovarian tumors, sex cord-stromal tumors, Meigs syndrome, CA-125, differential diagnosis, ovarian neoplasms

## Abstract

Sex cord-stromal tumors are rare ovarian neoplasms, with fibromas comprising approximately 4% and thecomas accounting for 0.5%–1% of all ovarian tumors. The occurrence of these tumors outside the ovaries is exceptionally rare and diagnostically challenging, often mimicking malignancy when associated with ascites, elevated CA-125 levels, or Meigs-like syndrome. This review aims to synthesize current knowledge on the histopathological, immunohistochemical, radiological, and molecular features of ovarian fibroma-thecoma group tumors and highlight their clinical relevance. We report two postmenopausal women with large abdominal masses located extraovarian: one in the broad ligament and the other adherent to the omentum and intestines. In the first case, markedly elevated CA-125, ascites, and pleural effusion initially suggested Meigs syndrome. The second case presented with an abdominal mass and ascites. Imaging studies indicated the possibility of malignant ovarian tumors in both patients, leading to surgical excision. Histopathological examination revealed spindle-to-oval tumor cells arranged in fascicular or storiform patterns, with focal lipid-rich theca-like cells. Immunohistochemical analysis showed that the tumors were positive for vimentin, WT1, progesterone receptor (PR), and variably for estrogen receptor (ER), CD56, inhibin, and calretinin, while being negative for markers of epithelial, melanocytic, and gastrointestinal stromal tumors. A review of the literature identified only 11 well-documented cases of extraovarian fibroma-thecoma group tumors, which most commonly arise in the broad ligament or pelvic cavity. These cases are frequently associated with ascites and elevated CA-125 levels and are often misdiagnosed preoperatively as malignant disease. Our cases underscore the importance of considering extraovarian fibromas and thecomas in the differential diagnosis of pelvic and abdominal masses presenting with similar features. Accurate pathological assessment can prevent unnecessary radical surgeries and promote more favorable patient outcomes.

## Introduction

Sex cord-stromal tumors are rare ovarian neoplasms [1]. According to the World Health Organization, these tumors are classified into pure stromal tumors, pure sex cord tumors, and mixed sex cord-stromal tumors, each originating from distinct ovarian cell types [2]. Fibromas account for only 4% of all ovarian tumors, while thecomas constitute 0.5%–1% [1]. Extraovarian occurrences of these tumors are exceptionally rare, with the majority of reported cases involving granulosa cell tumors [3]. Due to the rarity and diagnostic challenges associated with extraovarian fibroma-thecoma, diagnoses are often made post-surgery. Furthermore, extraovarian sex cord-stromal tumors are frequently misdiagnosed as ovarian cancers because of overlapping clinical and radiological features, complicating accurate pathological diagnosis [4].

This paper first provides a comprehensive literature review summarizing the current understanding of fibroma-thecoma tumors, emphasizing their histopathological and clinical characteristics. We then focus on their rare extraovarian occurrences, which remain underreported and diagnostically challenging. To illustrate these issues, we present two unusual cases of extraovarian fibrothecomas that posed significant diagnostic challenges, highlighting the critical role of accurate pathological assessment in preventing misdiagnosis and overtreatment.

## Materials and methods

### Histological processing and staining

Tissue samples were fixed in 10% neutral buffered formalin for 24–48 h, routinely processed, and embedded in paraffin. Sections were cut to a thickness of 3–4 µm and stained with hematoxylin and eosin (H&E). Immunohistochemistry was conducted using commercially available antibodies with a Shandon Sequenza Immunostaining Center. Additionally, special stains, including Sudan and reticulin, were performed.

### Radiological imaging

CT imaging was conducted utilizing a Siemens SOMATOM Definition Edge 128-slice CT scanner. Axial plane scans were obtained with a slice thickness of 3 mm, employing standard soft tissue and bone reconstruction algorithms. An experienced radiologist reviewed the images.

### Ethical statement

The Ethical Committee of the University Clinical Center Tuzla waived the requirement for ethics approval in accordance with institutional guidelines for case reports. Written informed consent was obtained from the patient for the publication of this case report and any associated images, in line with the procedure titled *“Hospital Policies and Procedures: Obtaining Patient Consent.”* This procedure includes a sub-document titled *“Informed Consent for Inclusion in Clinical Trials,”* which patients sign upon admission to the hospital.

## Case 1

The 56-year-old patient presented with complaints of lower abdominal swelling, intermittent abdominal pain lasting several days, dysuria, and irregular bowel movements. Her medical history included two pregnancies, and she had been in menopause for 15 years. Additionally, she was receiving medication for hypertension. Physical examination revealed a soft abdominal wall above the thorax, which was mildly tender upon palpation.

Ultrasound imaging identified a predominantly solid mass measuring 150 x 120 mm in the left adnexal structures, accompanied by free fluid in the Douglas pouch. The clinical working diagnosis was a left adnexal tumor with associated ascites. Given the mass’s size, solid composition, and presence of fluid, there was significant concern for potential malignancy. A consultation with a gynecological oncology team recommended surgical intervention.

A median laparotomy was performed, which revealed a solid, brownish tumor adherent to the omentum and intestines. An abdominal surgeon was subsequently consulted, and tumor resection was conducted alongside omentectomy, which included a 30 cm resection of the small intestine, followed by a T-T anastomosis. The excised specimen was sent for histopathological evaluation. The pelvic organs, including the uterus and adnexa, appeared normal and were preserved in situ. A drain was inserted into the Douglas pouch, and the abdominal cavity was irrigated prior to closing the incision in layers.

The specimen submitted to the pathology department comprised a resected segment of the small intestine measuring 220 mm, which contained a tumor measuring 150 mm × 140 mm × 90 mm on the adjacent omentum. Macroscopically, there was no evidence of infiltration into the wall of the small intestine. The tumor exhibited a smooth outer surface, while the cut surface displayed a variegated appearance, featuring a distinct area of white, solid, fibrous tissue surrounded by fatty, hemorrhagic, softened, and partially necrotic tissue.

**Figure 1.**
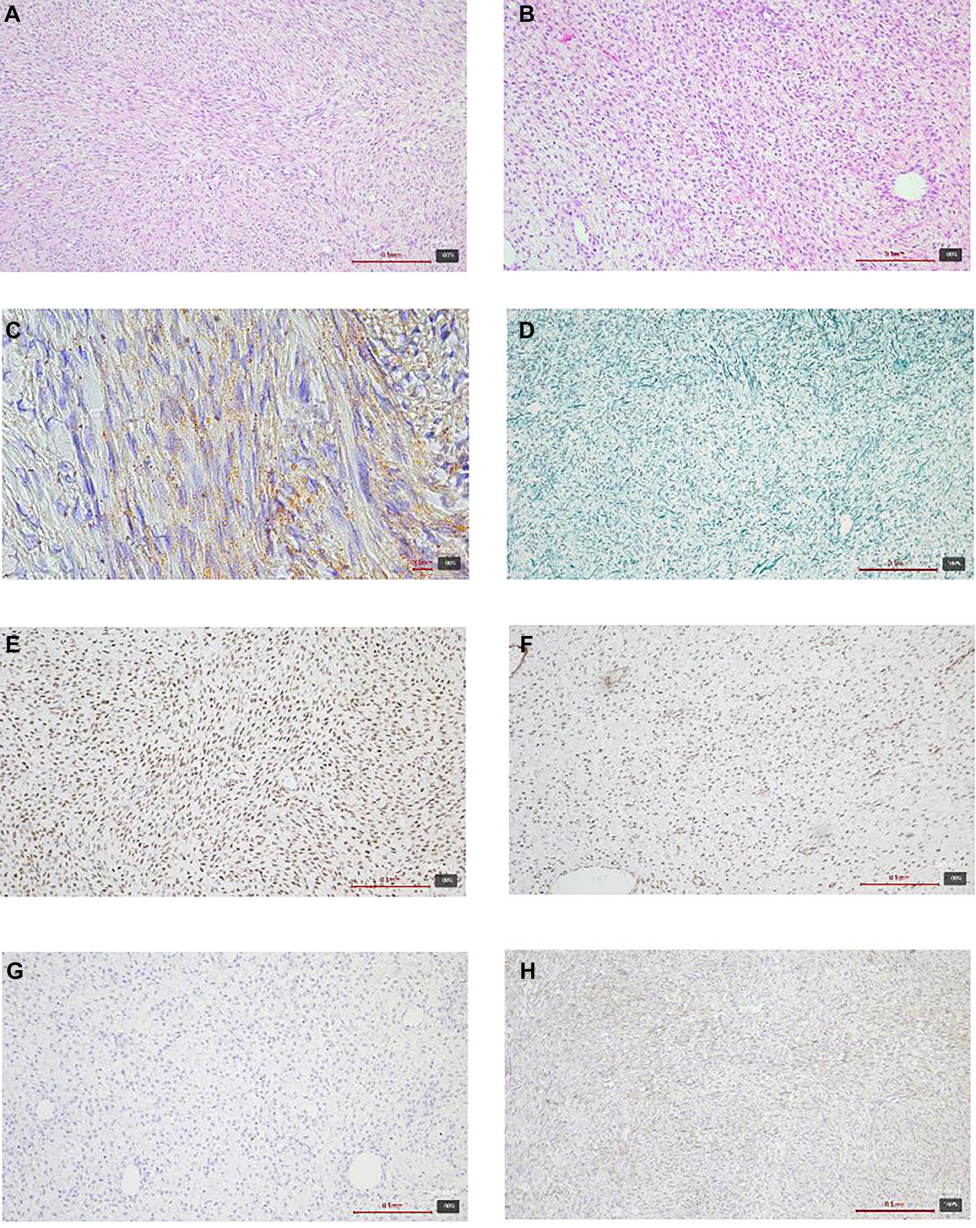
**Histologic and immunohistochemical features of an extraovarian fibrothecoma (case 1).** (A and B) Hematoxylin–eosin (H&E): Spindle to ovoid tumor cells in fascicular and storiform bundles with focal collagenized, hypocellular areas; (C) Sudan stain: Focal cytoplasmic lipid droplets in theca-like cells; (D) Reticulin stain: Delicate pericellular reticular network; (E) Progesterone receptor (PR): Nuclear positivity; (F) WT1: Diffuse nuclear positivity; (G) Desmin: Negative; (H) Calretinin: Focal positivity. Magnification 10×.

Microscopically, adjacent to the serosa of the small intestine displaying signs of chronic adhesive serositis, a cellular tumor was identified. The tumor exhibited ischemic necrosis and a fascicular and storiform growth pattern, consisting of relatively uniform cells with spindle-shaped to oval nuclei. Certain areas contained cells with lighter, more abundant cytoplasm, where small fat droplets were focalized, as demonstrated by Sudan staining. Reticulin staining revealed a delicate reticular framework, along with focal areas of hypocellular, collagenized connective tissue. The mitotic activity was low. Immunohistochemical analysis showed that the tumor cells were positive for vimentin, SMA, WT1, PR, and, focally, for ER, CD56, inhibin, and calretinin, while being negative for AE1/AE3, S-100 protein, desmin, CD34, CD117, Myo D1, and CD10. The Ki67 proliferative index reached up to 5%. No ovarian tissue was detected in the analyzed tumor sections (Figure 1).

In the differential diagnosis, other sex cord-stromal tumors, including fibroma, thecoma, and adult granulosa cell tumor (AGCT), were considered, along with soft tissue tumors such as gastrointestinal stromal tumor (GIST), leiomyoma/leiomyosarcoma, and solitary fibrous tumor. However, following a comprehensive microscopic analysis, immunohistochemical testing, and evaluation of the clinical presentation, a diagnosis of extraovarian fibrothecoma was established.

The patient’s recovery was uneventful, characterized by proper wound healing and the absence of complications. Drains were removed on the fifth postoperative day, and she was discharged in good condition, pain-free, and active. A documented follow-up examination by a gynecologist revealed no abnormalities; tumor markers were within the normal range, with CA-125 at 8.2 U/mL and CEA at 0.7 ng/mL. No additional gynecological pathologies were noted. The patient continued to receive medical supervision for unrelated cardiovascular conditions, including aortic valve stenosis and insufficiency, as well as an aneurysm of the aortic root and ascending aorta.

## Case 2

**Figure 2.**
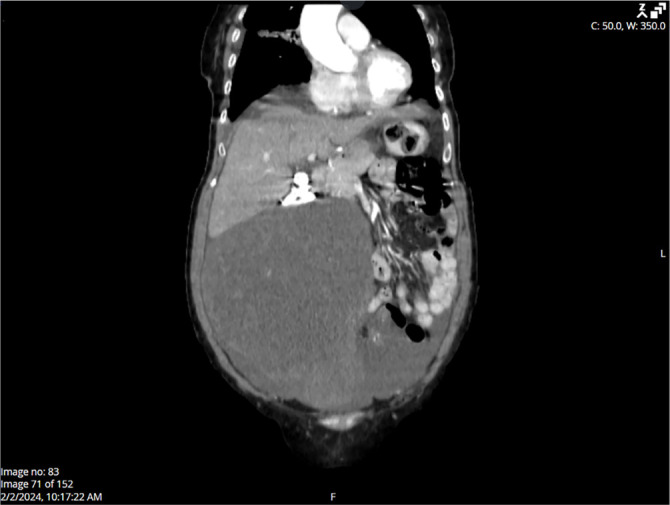
**CT scan displaying a large oval solid lesion originating from the medial margin of the liver in the right hemiabdomen, extending toward the right ovarian fossa.** The lesion exerts pressure on the surrounding vasculature and causes distortion of the right anterior abdominal wall.

The 75-year-old patient presented with a palpable abdominal mass. Her obstetric history included four births, and her last menstrual period occurred 20 years ago. Laboratory markers included CA-125 at 551.6 U/mL, CA 19–9 at 2.76 U/mL, and CEA at 1.09 ng/mL. Ultrasound findings indicated free fluid in the Douglas space and a solid, heterogeneous mass extending nearly to the xiphoid process. CT imaging confirmed a large cystic-solid tumor originating from the right ovary, extending cranially into the right hemiabdomen, displacing intestinal loops and deforming the right abdominal wall (Figure 2). Significant ascites was noted around the liver, spleen, and throughout the abdomen and pelvis. The gallbladder contained calculi; however, no other significant abnormalities were observed in the liver, pancreas, kidneys, or other abdominal organs. The left ovary was not visualized, and the urinary bladder was compressed by the tumor. Additionally, minimal pleural effusion was noted in the right lung. The thoracic surgeon concluded that urgent surgical intervention was unnecessary, as the pleural effusion did not significantly affect respiratory function, allowing the patient to proceed with gynecological surgery. The clinical working diagnosis was a right ovarian tumor with minimal right-sided pleural effusion.

A midline laparotomy revealed 1500 mL of free fluid and a large tumor mass occupying the abdominal cavity. The tumor was excised, and a total hysterectomy with bilateral adnexectomy was performed. The uterus and right adnexa appeared normal, while the left adnexa were adherent to the abdominal wall, although they were not in continuity with the tumor. Additionally, an omental biopsy and cytological samples were obtained.

The pathology department received a uterus with adnexa, accompanied by a separately submitted mass measuring 250 × 200 × 120 mm. The mass exhibited a grayish-white color with a smooth surface and a prominent vascular pattern. Focal yellowish discoloration was observed, along with an area characterized by an uneven, torn surface. Upon sectioning, the mass appeared solid, grayish, and fibrous, with focal glassy regions and areas of hemorrhage. Biopsy of the omentum and cytological analysis of the abdominal sample yielded no pathological findings or tumor deposits.

**Figure 3.**
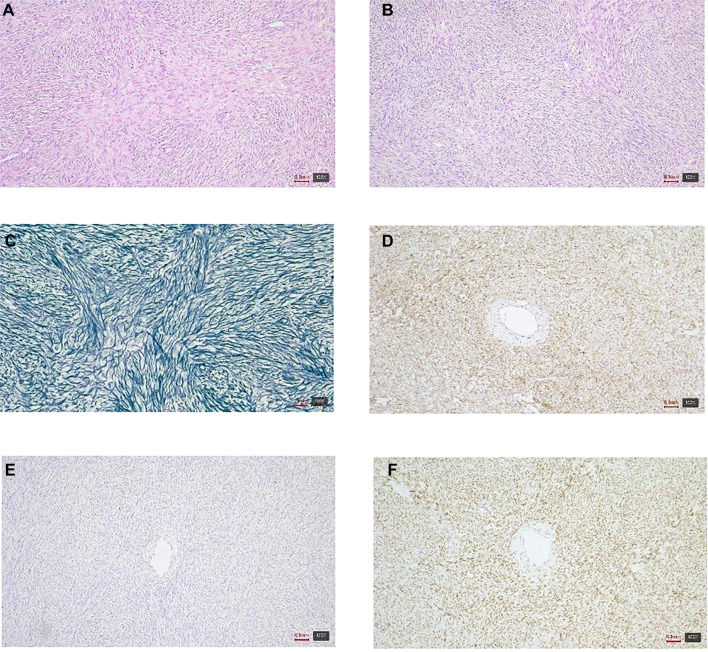
**Histologic and immunohistochemical features of an extraovarian fibrothecoma (case 2).** (A and B) Hematoxylin–eosin (H&E): Spindle to polygonal tumor cells arranged in bundles and whorled patterns with variable cellularity; (C) Reticulin stain: Reticular fibers outlining individual cells and small groups; (D) CD56: Diffuse membranous positivity; (E) Desmin: Negative; (F) FOXL2: Nuclear positivity. Magnification 10×.

Microscopic analysis of the mass identified a tumor characterized by cellular arrangements in bundles and whorled patterns, featuring oval to slightly elongated, mildly chromatic nuclei and moderately abundant eosinophilic cytoplasm (Figure 3). Another component of the tumor exhibited diffusely arranged cells with round to polygonal nuclei and a more abundant, lighter cytoplasm. Occasionally, thin-walled vascular spaces were observed within the tumor.

Immunohistochemical analysis indicated that the tumor cells were positive for WT1, PR, FOXL2, CD56, vimentin, and showed focal positivity for ER, inhibin, calretinin, and SMA. In contrast, the cells were negative for desmin, S-100, p53, CD34, HMB45, CD10, and EMA. Reticulin staining demonstrated a reticular network surrounding individual and clustered cells, while Sudan staining revealed lipid droplets within the cytoplasm of select cells. No mitotic figures were detected, and the Ki67 proliferative index was approximately 3%.

Although the tumor morphologically resembled a member of the sex cord tumor group, a comprehensive range of immunohistochemical analyses was conducted to exclude other similar spindle-cell tumors. Based on the clinical presentation and microscopic findings, a diagnosis of extraovarian fibrothecoma was established.

Postoperatively, the patient was placed on BIPAP ventilation, gradually awakened, and extubated while remaining conscious and breathing spontaneously with oxygen support via HFNC. A follow-up chest X-ray indicated progression of infiltrative shadowing in the right lung and an increase in pleural effusion. The thoracic surgeon subsequently drained 2000 mL of serous fluid. The patient received treatment with antibiotics and anticoagulants and was discharged after 8 days.

One month following surgery, a follow-up examination by the thoracic surgeon, including a chest X-ray, confirmed regression of the pleural effusion. Two months postoperatively, a gynecological examination was performed, yielding unremarkable results. No further gynecological follow-up visits were recorded. The clinical course during this period was uneventful.

## Fibroma-thecoma tumors

**Figure 4.**
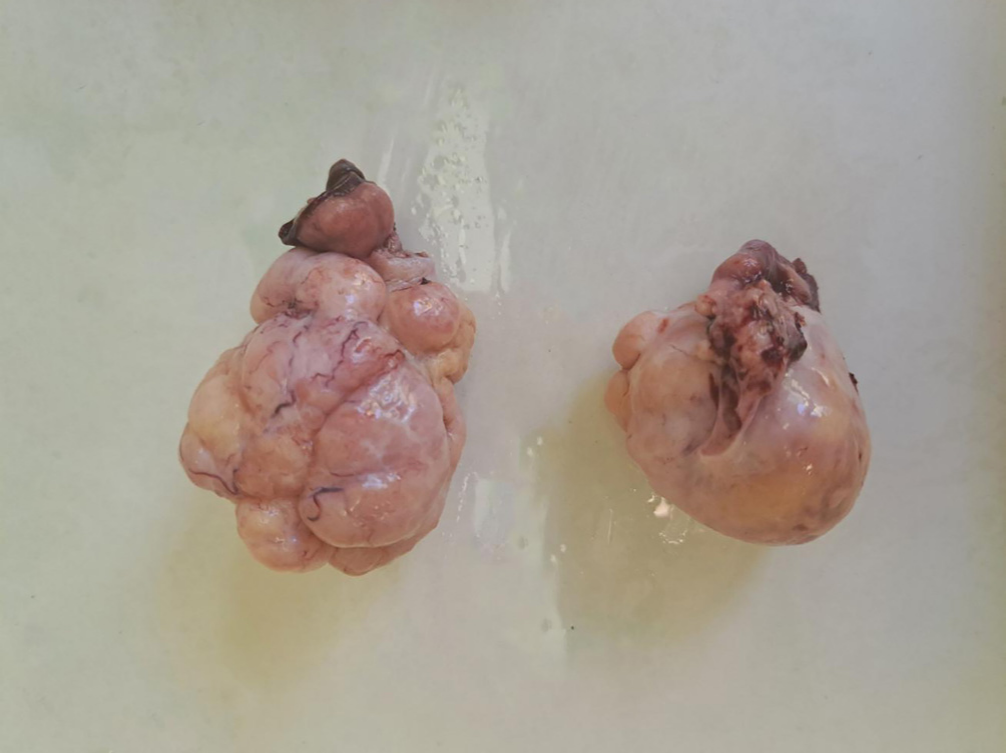
**Ovarian fibroma showing a nodular, solid, white–tan surface.** Gross photograph of two resected specimens demonstrating lobulated, firm masses with smooth, glistening external surfaces and prominent nodularity, without obvious cystic change on the surface.

**Figure 5.**
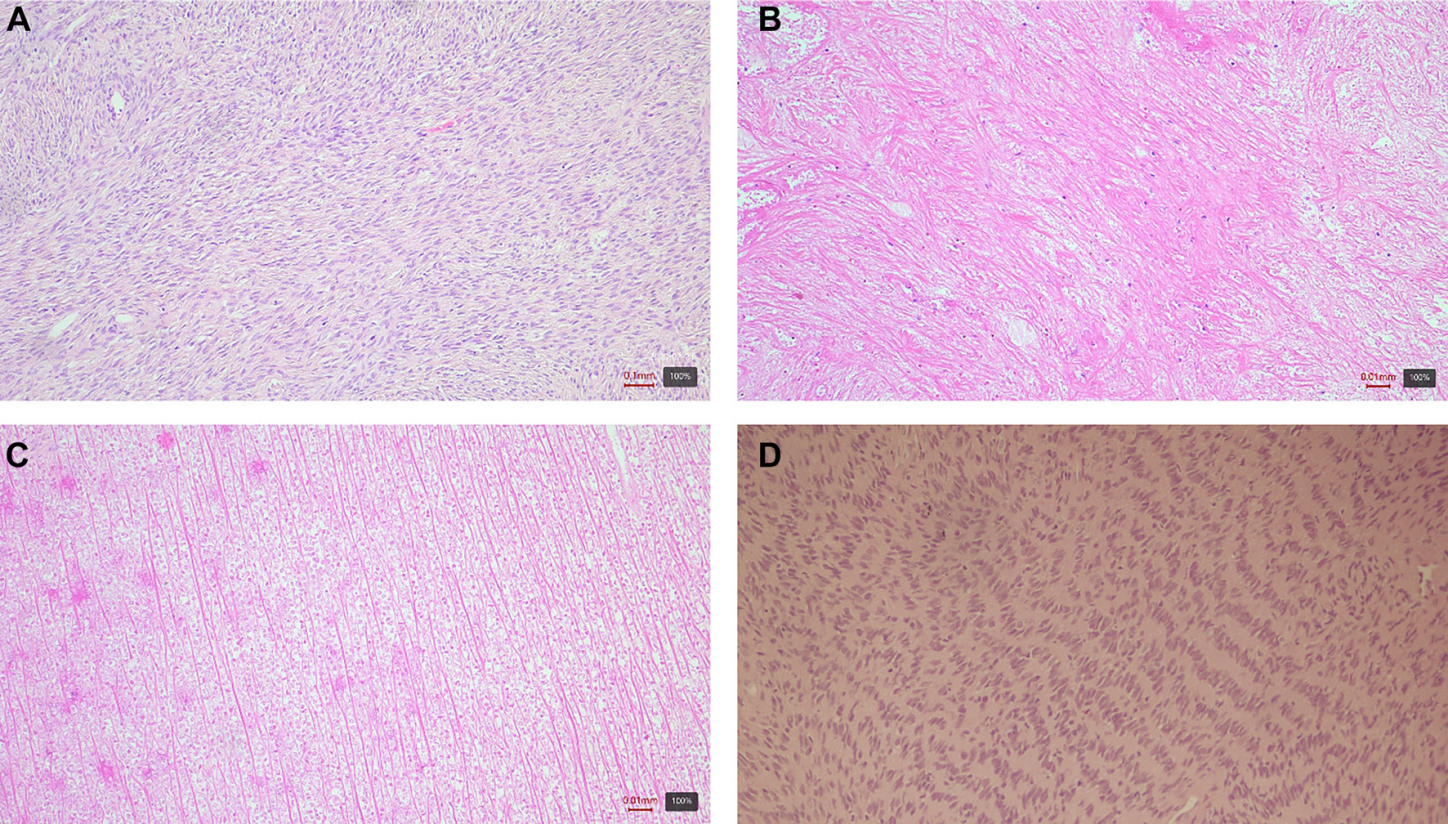
**Histopathological features of ovarian fibroma.** (A) H&E staining showing cytologically bland spindle cells arranged in interlacing or storiform fascicles; (B) Hyalinized plaques, present in approximately one-third of cases; (C) Areas of ischemic necrosis, which should not be misinterpreted as a sign of malignancy; (D) Cellular fibroma with increased cellularity but lacking nuclear atypia. All images at 5× magnification; scale bar = 100 µm. Abbreviation: H&E: Hematoxylin–eosin.

Ovarian fibromas are predominantly benign, unilateral, hormonally inactive tumors that typically present as firm, white nodules upon sectioning (Figure 4) [5]. Histologically, they are characterized by bland spindle cells arranged in interlacing or storiform fascicles, and most cases do not present diagnostic challenges (Figure 5A) [6]. Fibromas are generally smaller than thecomas and exhibit a diffuse growth pattern. Tumor cells are usually embedded within a fibrous stroma, which may be edematous in a minority of cases [7].

Common features of ovarian fibromas include focal calcifications, hyalinized plaques (Figure 5B) (seen in approximately 37% of cases), and approximately third of the tumors show increased vascularity, and cystic degeneration. These characteristics can resemble those of sclerosing stromal tumors. Cystic degeneration may mimic epithelial–stromal tumors macroscopically; however, the absence of epithelial lining on microscopy clarifies the diagnosis [5]. Although most fibromas are histologically uniform, some display alternating zones of edema, hypercellularity, or vascularity, which can also mimic sclerosing stromal tumors. When present, necrosis is typically ischemic in nature and should not be mistaken for an indicator of malignancy (Figure 5C). Notably, up to one-third of fibromas may exhibit more abundant cytoplasm than typically expected [7].

Fibromas may contain luteinized cells, although these do not possess the fibroblast-rich or vacuolated stroma characteristic of sclerosing stromal tumors. The presence of luteinized cells is usually noted only when clinically significant, such as in androgen-secreting tumors [5].

Challenges may arise when tumors exhibit increased cellularity, mitotic activity, or cytologic atypia (Figure 5D). Cellular fibromas (CFs) are characterized by increased cellularity, comparable to diffuse adult cell granulosa tumors, but lack atypia and elevated mitotic activity, representing approximately 10% of all ovarian fibromas [5, 8]. Compared to conventional fibromas, CFs are larger, possess a softer consistency, and exhibit a tan to yellow cut surface [8]. While they often contain areas of conventional fibroma, they are less frequently associated with edema and hyalinized plaques. Despite their dense cellularity, CFs display only minimal cytologic atypia, a crucial criterion for diagnosis. Increased mitotic activity, however, is not uncommon. The histological criteria established in 1981 by Prat and Scully are widely employed to distinguish CFs from ovarian fibrosarcomas, utilizing a threshold of three mitoses per 10 high-power fields (HPFs) [9]. CFs exhibiting high mitotic rates but bland nuclei should not be misclassified as fibrosarcomas, as this may result from an overemphasis on mitotic counts and insufficient recognition of nuclear atypia. If the mitotic count exceeds four per 10 HPFs without accompanying atypia, the lesion is classified as a mitotically active CF (MACF). These tumors are generally not associated with poor prognosis; however, recurrence has been reported in cases of incomplete excision, tumor rupture, or surface adhesions [5].

MACFs present several distinctive features compared to conventional CFs [9]. They typically occur in younger patients (mean age 41 vs 51 years), are slightly larger (mean diameter 9.4 cm vs 8.0 cm), yet exhibit similar clinical presentations and gross pathological characteristics. All tumors were unilateral and predominantly solid, with occasional cystic areas. A minority in both groups demonstrated ovarian surface involvement or limited extraovarian spread. Notably, despite increased mitotic activity (mean 6.7 mitoses/10 HPFs, range 4–19), MACFs lack nuclear atypia. Follow-up data from 18 cases indicated no recurrence or adverse clinical outcomes, reinforcing the idea that mitotic activity alone, in the absence of atypia, does not suggest malignancy.

In cases of prominent edema, tumors may present as soft masses, which can result in their misclassification as thecomas [5]. Severe edema may also be associated with Meigs syndrome. Additionally, bilateral calcified fibromas are less frequently linked to Gorlin syndrome (basal cell nevus syndrome), wherein pathologists can play a crucial role in identifying the syndrome prior to the emergence of overt clinical manifestations.

### Ovarian thecoma

**Figure 6.**
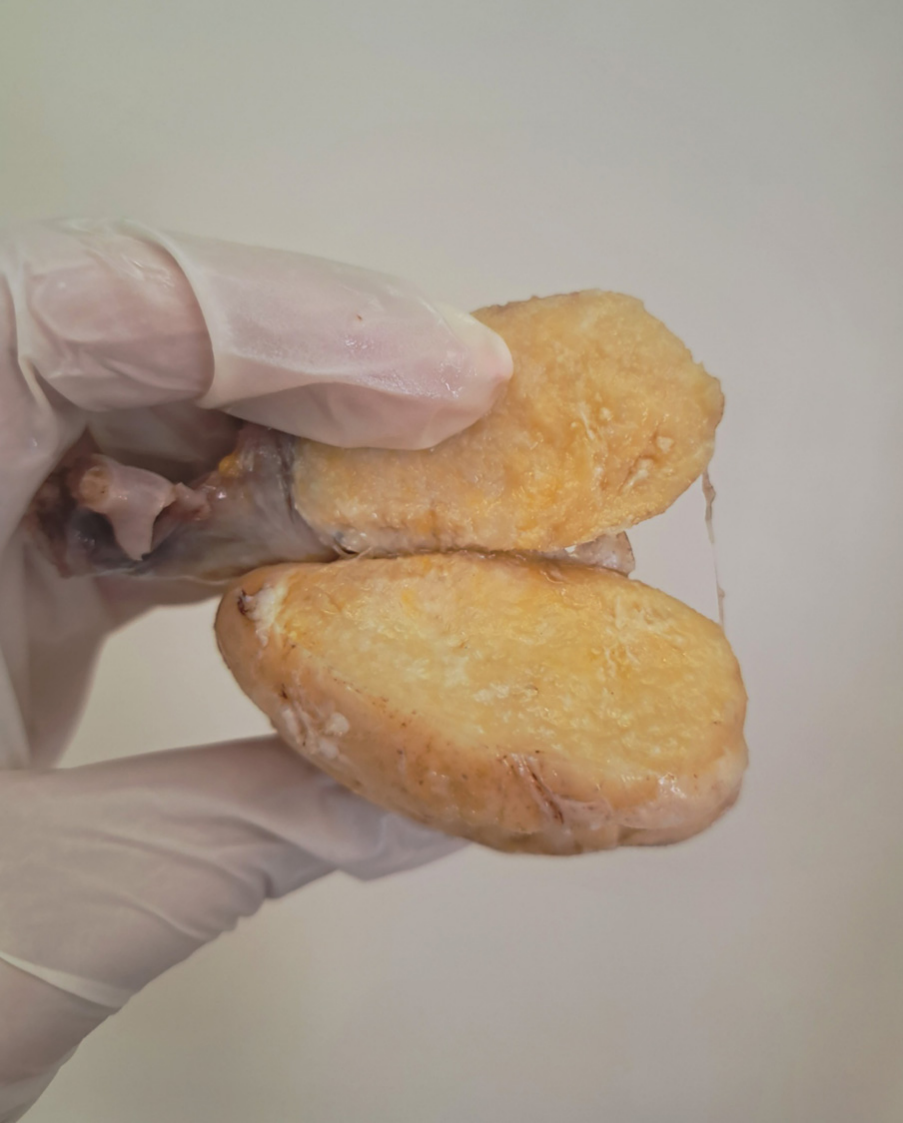
**Ovarian thecoma.** Gross specimen demonstrating a solid, well-circumscribed mass with a lobulated, bright yellow cut surface, consistent with lipid-rich theca cell composition.

Thecomas are rare, potentially estrogen-producing ovarian tumors characterized by eosinophilic cytoplasm that resembles theca cells [6]. Most thecomas are unilateral, solid tumors; however, some display a mixed solid and cystic architecture [10–12]. Upon sectioning, these tumors exhibit a range of consistencies from firm to stony hard, with lobulated surfaces and variable coloration, ranging from chalky white to bright yellow (Figure 6). Calcifications may be present, while hemorrhage and necrosis are uncommon and typically observed only in isolated cases.

**Figure 7.**
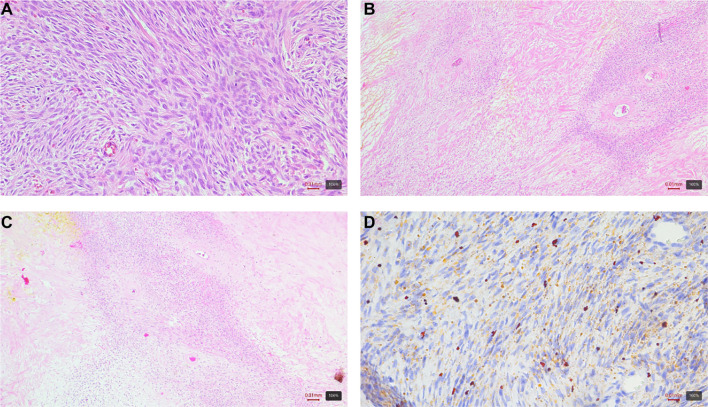
**Ovarian thecoma key microscopic features.** (A) Densely cellular fascicles of thecomatous cells in storiform/whorled pattern, visible only in the central part of the image (H&E, 20×); (B and C) Prominent stromal hyalinization with hyalinized plaques typical for thecoma (H&E, 10×); (D) Cytoplasmic lipid demonstrated by positive Sudan staining (20×).

Microscopically, thecomas exhibit architectural variability, which can range from densely cellular fascicles arranged in storiform or whorled patterns to more edematous and hypocellular forms (Figure 7A) [7]. Tumor cells typically replace the ovarian medulla and may compress or obliterate the cortex. Stromal hyalinization is a common characteristic, with hyalinized plaques frequently observed in thecomas, although they can also be found in fibromas (Figure 7B and 7C). Some thecomas may present significant sclerosis or calcification, particularly in younger patients [5]. Cytoplasmic vacuolation, indicative of lipid content, occurs in approximately one-third of thecomas; however, true lipid positivity is confirmed in fewer cases, suggesting that these vacuoles are not consistently lipid-rich (Figure 7D) [7]. Cytologic atypia and mitotic activity are generally minimal, although bizarre nuclei may be present in exceptional cases [5]. Additionally, thecomas are notably enriched in mast cells, with one study reporting densities ranging from 100 to 330 mast cells per 100 HPFs, as detected by Giemsa staining [10].

Rare instances of malignant thecomas have been documented [11]. In two cases, one presented as a unilocular cystic mass with an irregular solid component and coarse margins, resulting in a misdiagnosis of cystadenocarcinoma on MRI. The other case manifested as a large solid mass with multiple non-enhancing areas and moderate ascites on CT, initially misidentified as malignant epithelial ovarian carcinoma.

### Ovarian fibrothecoma

**Figure 8.**
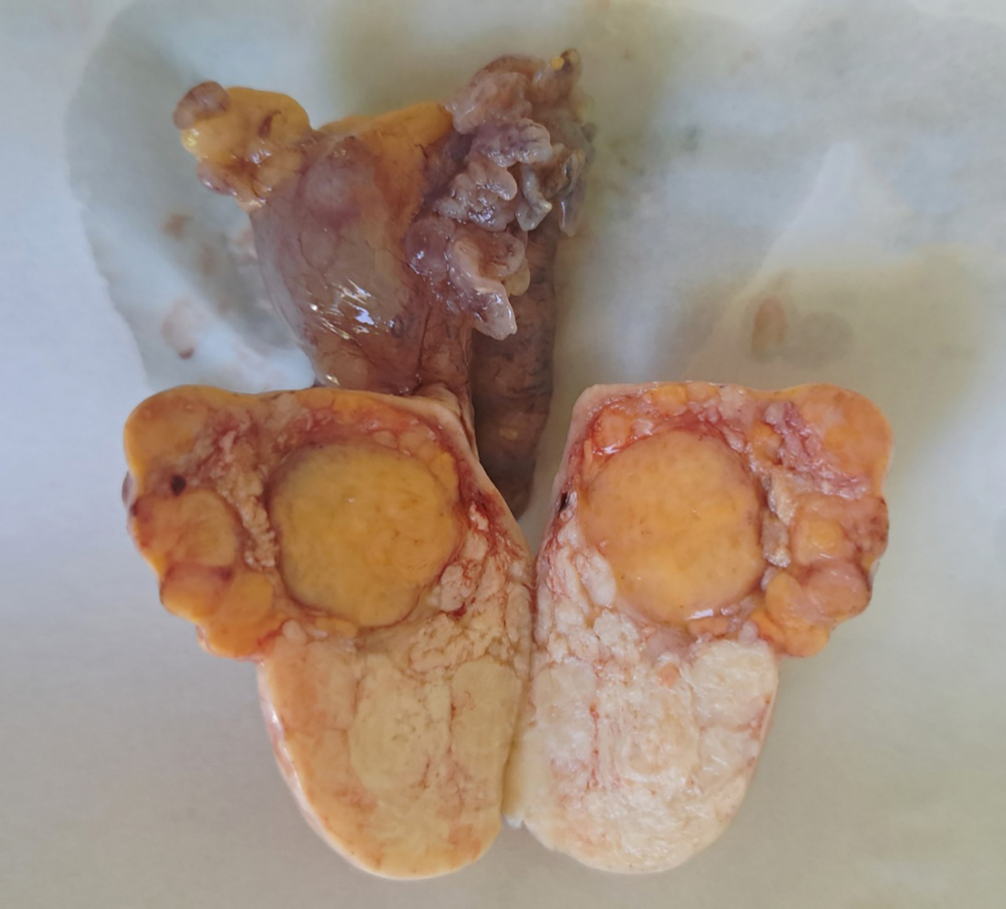
**Ovarian fibrothecoma gross appearance.** Bisected, well-circumscribed mass with a lobulated cut surface showing soft, yellow thecomatous nodules set within firm, white fibrous tissue—illustrating a fibromatous background with focal thecomatous areas consistent with fibrothecoma.

When both fibroma and thecoma components coexist in a tumor, classification should be determined by the dominant morphological component. The diagnosis of thecoma is preferred when the tumor predominantly features cells with abundant vacuolated or luteinized cytoplasm, accompanied by hyaline plaques between nodular cell clusters. In contrast, tumors that exhibit a fibromatous appearance with only focal thecomatous characteristics are typically classified as fibrothecomas (Figure 8) [13]. Although the term “fibrothecoma” was removed from the WHO classification in 2014—having previously been included in the “unclassified” group in 2003 [2]—it remains widely utilized in contemporary studies [12, 14–18]. The designation “fibrothecoma” is not clinically problematic, given the indolent behavior exhibited by both entities [5].

### Immunohistochemistry

Immunohistochemical analysis reveals that fibromas and CFs exhibit limited immunoreactivity for α-inhibin, often being negative or only focally positive [13, 19]. In comparison, calretinin is more frequently expressed and is regarded as a more reliable marker for these tumors. Conversely, thecomas typically show strong and diffuse positivity for both α-inhibin and calretinin, aiding in their differentiation.

All ovarian stromal tumors, including fibromas and CFs, exhibit strong cytoplasmic positivity for CD56 and nuclear positivity for WT1, progesterone receptor (PR), and estrogen receptor beta (ER-β) [20]. Additionally, they are typically positive for FOXL2 [21]. These tumors show variable positivity for smooth muscle actin (SMA) and muscle-specific actin (MSA), while consistently negative for CD34 and S-100, which confirms their stromal origin and aids in differentiating them from other spindle cell tumors of the ovary [20, 22]. Notably, positive nuclear and cytoplasmic S-100 staining has been reported in fibromas and fibrothecomas. Furthermore, fibromas are negative for CD99, CD10, cytokeratins, and epithelial membrane antigen (EMA) [13, 23, 24]. Overview of immunohistochemical findings in two fibrothecoma cases presented in this study and their differential diagnoses is given in Table 1.

### Clinical presentation and demographics

These tumors predominantly affect women around the age of 51 [10–12, 14, 17, 25, 26], with a significant majority being postmenopausal [11, 14, 25, 26]. They are frequently identified due to symptoms such as pain, abdominal distension, or vaginal bleeding [15–17, 26], although some studies indicate that 30%–64% of cases are discovered incidentally [10, 25]. Additionally, certain cases are reported to be identified following tumor torsion [12, 14].

While thecomas and fibrothecomas can induce hyperestrogenism, this occurrence is less frequent compared to granulosa cell tumors [27]. Clinically, these tumors may manifest symptoms akin to those of granulosa cell tumors, particularly abnormal uterine bleeding. Luteinized thecomas, characterized by steroid-producing cells, are more commonly observed in younger patients and exhibit estrogenic effects in approximately half of the cases. Virilization is a rare complication, occurring in around 11% of cases.

In a study involving 24 patients with thecomas who underwent hysterectomy for endometrial pathology, 13 (54.2%) were diagnosed with simple hyperplasia, 5 (20.8%) with endometrial adenocarcinoma, and 6 (25%) with endometrial polyps, underscoring the potential hormonal activity of these tumors [10]. Another investigation focused solely on thecomas, revealing endometrial hyperplasia in 2 out of 19 cases (10.5%) and endometrial carcinoma in 1 case (5.2%), both attributed to elevated estrogen levels [11]. Furthermore, two studies examining fibrothecomas reported endometrial hyperplasia rates of 11% and 35%, respectively [15, 16].

Approximately half of the women studied presented with fluid accumulation in the Douglas pouch [28]. Ascites was observed in 11% to 67% of cases [12, 14–17, 25, 26], while elevated CA-125 levels were reported in 11% to 61% of patients diagnosed with ovarian fibromas-thecomas [11, 12, 14, 15, 17, 18, 25, 26]. Notably, larger tumor size (10 cm or more), the presence of ascites, hydrothorax, and Meigs syndrome were more frequently associated with elevated CA-125 levels. Among these characteristics, large tumor size and ascites exhibited the strongest correlation with increased CA-125, suggesting that these factors may contribute to elevated serum levels despite the benign nature of the tumor.

Meigs syndrome is characterized by benign ovarian tumors accompanied by ascites and pleural effusion, typically presenting with dyspnea, dry cough, and painful abdominal distension [29]. This syndrome occurs in approximately 1% of ovarian tumors and often mimics malignant disease, complicating accurate diagnosis. Tumors associated with Meigs syndrome include fibromas-thecomas, struma ovarii, sclerosing stromal tumors, and granulosa cell tumors [30]. Ascites and pleural effusion generally resolve following surgical removal of the tumor. Reports of Meigs syndrome in case series range from 1% to 11% [14, 17, 25, 26]. The syndrome can be classified into classic, non-classic, Demons-Meigs, and pseudo-Meigs forms [30]. The classic form encompasses a benign ovarian tumor, ascites, pleural effusion, and resolution after surgery. The non-classic variant includes tumors of the Fallopian tube or broad ligament. Pseudo-Meigs refers to similar findings associated with non-ovarian tumors, whether benign or malignant. Incomplete Meigs syndrome is characterized by the presence of either ascites or pleural effusion, with classification dependent on tumor type and biological behavior.

### Extraovarian fibrothecomas

A literature search was conducted using Web of Science, Scopus, and PubMed to identify reported cases of fibroma-thecoma group tumors in both ovarian and extraovarian locations. The search encompassed all articles published up to December 2024. The keywords utilized included “fibroma,” “thecoma,” “fibrothecoma,” “sex cord-stromal tumor,” “ovarian,” and “extraovarian.” Only English-language case reports were considered, while articles lacking sufficient clinical, imaging, or pathological details were excluded. Relevant data were extracted and summarized, including patient age, tumor location, size, clinical presentation, histopathological findings, and reported outcomes. A total of sixteen articles were identified, of which five were excluded due to being non-English or lacking complete text.

**Table 1:** Overview of immunohistochemical findings in two cases and their differential diagnoses

**Tumor Entity**	**SMA**	**WT1**	**PR**	**Inhibin**	**Calretinin**	**AE1/AE3**	**S-100**	**Desmin**	**CD34**	**CD117**	**ER**	**CD10**	**Ki-67**	**FOXL2 (IHC)**	**CD56**	**EMA**	**p53**	**Reticulin**	**Sudan Stain**	**Reference**
Case 1: Fibrothecoma	+	+	+	±	±	−	−	−	−	−	±	−	∼5%	NA	**±**	−	Wild type	+	+	
Case 2: Fibrothecoma	±	+	+	±	±	NA	−	−	−	NA	±	−	∼3%	+	+	−	Wild type	+*	+	
Fibroma	+	+	±	±	±	−	−	−	−	−	±	−	Low	±	±	−	Wild type	+	−	[2, 22]
Thecoma	+	+	+	+	+	−	−	−	−	−	+	−	Low	±	±	−	Wild type	+	+	[2, 22]
Fibro-sarcoma	±	+	±	±	±	−	−	−	±	−	±	±	High	−	−	−	+	±	−	[55, 56]
AGCT	±	±	+	+	+	±	±	±	±	−	+	±	Variable	+	±	−	Wild type	±	−	[2, 13, 24]
SLCT	+ (in Leydig cells)	+	±	+	+	±	−	± (positive in Leydig cells)	−	−	±	±	Variable	±	±	−	Wild type	± (“pericellular” or “tubule-encircling” pattern in sertoli cells, absent in leydig cells)	−	[2, 13, 24]
Ovarian Leiomyoma	+	±	+	−	−	−	−	+	−	−	+	±	Low	−	−	−	Wild type	±	−	[57, 58]
ESS	±	±	+	− (positive in areas of sex cord differentiation)	− (positive in areas of sex cord differentiation)	±	±	±	±	−	+	+	Variable	−	+	−	Wild type	±	−	[2, 59−61]
GIST	±	−	−	−	−	−	−	−	+	+	−	−	Variable	−	−	−	±	−	−	[62]

+: Positive; **±**: Focal or variable positivity; −: Negative; *individual and grouped cells. Variable expression varies among cases. Abbreviations: SMA: Smooth muscle actin; WT1: Wilms tumor 1; PR: Progesterone receptor; AE1/AE3: Cytokeratin AE1/AE3; ER: Estrogen receptor; IHC: Immunohistochemistry; EMA: Epithelial membrane antigen; NA: Not applicable; AGCT: Adult granulosa cell tumor; SLCT: Sertoli–Leydig cell tumor; ESS: Endometrial stromal sarcoma; GIST: Gastrointestinal stromal tumor.

In the analysis of 11 cases, the following findings were observed: 4 fibrothecomas (including one with sex cord elements) [31–34], 3 pure thecomas [35–37], and 4 fibromas (including two with minor sex cord elements and one containing steroid cells) [3, 4, 38, 39]. A summary of the analyzed cases is presented in Table 2.

**Table 2:** Cases of extraovarian stromal tumors

**Case**	**Entity**	**Age (y)**	**Size (cm)**	**Location**	**Tumor markers**	**Clinical presentation**	**Author(s)**	
1	Fibrothecoma	62	18	Left retroperitoneum	CA125: 1291 U/mL	Gradual abdominal distension, ascites, abdominal mass	Roberts et al. (2012) [31]	
2	Fibrothecoma	44	10	Pelvic pouch	NA	Recurrent superficial thrombophlebitis, incidental asymptomatic pelvic mass finding	Honore et al. (1985) [32]	
3	Fibrothecoma	83	13	Sacrouterine ligament	NA	Urinary incontinence	Geiger et al. (2020) [34]	
4	Fibrothecoma with minor sex cord elements	45	4	Broad ligament	Normal tumor markers	Pain and deterioration over 2 months, anemia	Chen et al. (2023) [33]	
5	Thecomas	14	16	Pelvic mass	CA125: 157 U/mL	Acute onset	Aubert et al. (2022) [35]	
6	Thecoma	70	20	Right broad ligament	Normal tumor markers	Palpable large mass, ascites	Lin et al. (1987) [36]	
7	Thecoma	32	11	Left broad ligament	NA	Acute abdominal pain	Kurtoglu et al. (2014) [37]	
8	Cellular fibroma	84	8.5	Pelvic pouch	Normal tumor markers	Vaginal bleeding	Kim (2022) [4]	
9	Fibroma with minor sex cord elements	66	15	Left broad ligament	CA125: 99.49 U/mL, CA19-9: 41.53 U/mL	Incidental finding	Omori et al. (2017) [3]	
10	Fibroma with minor sex cord elements	41	27	Sigmoid mesocolon	CA125: 51.5 U/mL	Gradual abdominal distension	Pohekar et al. (2024) [38]	
11	Fibroma and steroid cells	56	7.4	Pelvic mass	NA	Incidental pelvic mass finding	Wong and McCluggage (2019) [39]	

Abbreviations: NA: Not available; CA 125: Cancer antigen-125; CA 19-9: Carbohydrate antigen 19-9.

The reported age range of affected patients was 32–84 years, with a notable case involving a 14-year-old girl. Tumors were identified in various locations: three originated from the broad ligament, one from the sigmoid mesocolon, two from the retroperitoneum, one from the uterine-rectal ligament, two were located in the pelvic pouch, and two were classified as pelvic masses without specific localization. In our series, one tumor was found in the broad ligament, while another was adherent to the omentum and intestines. In the literature, three cases were identified incidentally. Other cases exhibited symptoms such as vaginal bleeding (one case) and urinary incontinence (one case), while three additional cases presented with gradual abdominal distension. A 14-year-old girl experienced an acute onset of symptoms, and one woman presented with acute abdominal pain due to torsion. None of the initial clinical diagnoses in these cases included extraovarian fibroma-thecoma. In our cases, one tumor presented as a palpable, asymptomatic mass, while the other had a shorter symptomatic duration. Tumor sizes varied from 4 to 27 cm, with our cases measuring 15 cm and 25 cm.

Tumor markers were assessed in six patients, four of whom exhibited elevated levels of CA-125, including one case with a level of 1291 U/mL. In one of our patients, the CA-125 level was more than tenfold above the reference range, which correlated with ascites and hydrothorax. Although sex cord stromal tumors are recognized for their hormonal activity and potential to cause hormonal imbalances [6], none of the reported patients displayed such symptoms. The 14-year-old girl exhibited elevated dehydroepiandrosterone sulfate levels, but without any other hormonal disturbances.

These findings underscore the generally benign nature of extraovarian fibroma-thecomas, even in instances characterized by ascites and elevated CA-125 levels. One patient exhibited a clinical presentation suggestive of Meigs syndrome [29]. Although the initial diagnosis aligned with Meigs syndrome, the hydrothorax did not spontaneously resolve following tumor removal; instead, it persisted and progressed, ultimately necessitating drainage. This atypical progression raises doubts about whether this case truly exemplifies classic Meigs syndrome. It is more accurately classified within the spectrum of non-classic Meigs syndrome, as the tumor was extraovarian [30]. Furthermore, the pleural effusion did not fully resolve post-resection, diverging from established diagnostic criteria.

The differentiation between fibroma and thecoma in extraovarian tumors presents significant challenges due to overlapping morphological characteristics, particularly in cases featuring mixed spindle and lipid-rich cells. This complexity warrants the use of the term “fibrothecoma” in our report. Additionally, Giemsa staining has been highlighted as an effective technique for identifying mast cells, which are frequently observed in thecomas [10]. Although we did not utilize Giemsa staining in our analysis, our diagnosis was based on a comprehensive evaluation of histological architecture and immunohistochemical findings.

**Figure 9.**
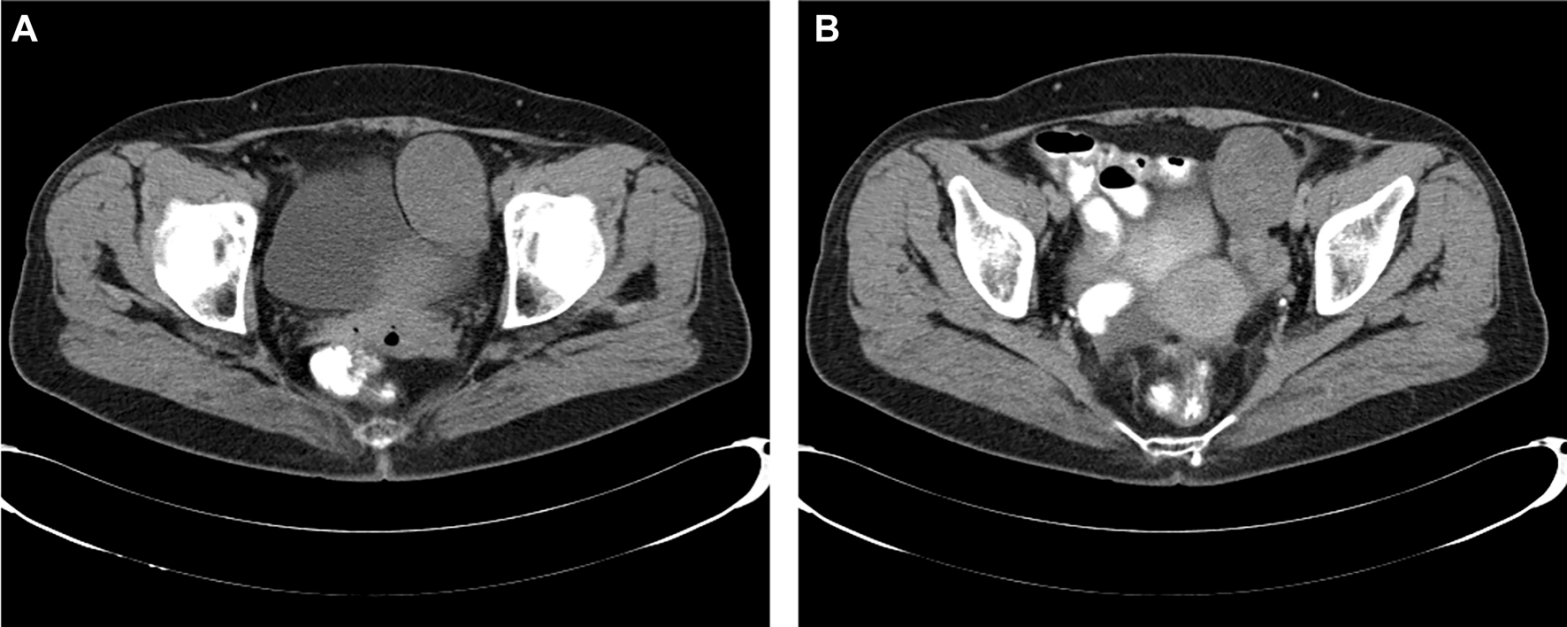
**Ovarian fibroma on CT.** Axial pelvic CT in pre-contrast (A) and post-contrast (B) phases shows a well-defined, lobulated left adnexal mass (50 × 42 mm) contiguous with the left ovary; the lesion demonstrates heterogeneous attenuation from thick fluid to soft-tissue density and lacks post-contrast enhancement—imaging features typical of the fibroma–thecoma group. Histology confirmed an ovarian fibroma.

To the best of our knowledge, this is the first documented case of an extraovarian fibrothecoma presenting with this specific constellation of findings. This underscores the necessity for broader consideration of this entity in comparable clinical contexts.

### Imaging and diagnostic work-up

Ultrasonography is frequently employed as the primary imaging modality for ovarian fibroma-thecomas. This technique typically reveals solid, hypoechoic, round or oval masses characterized by smooth borders, posterior wall attenuation, and a distinct echogenic pattern, which often overlaps with features of other pelvic masses [14, 40]. While these tumors usually exhibit a characteristic ultrasound appearance, atypical findings can complicate the differentiation from malignant tumors [17]. The Risk of Malignancy Index (RMI), which incorporates ultrasound features, menopausal status, and CA-125 levels, serves to distinguish benign from malignant adnexal masses; however, its accuracy for ovarian fibroma-thecomas is limited. In one study, 23.3% of cases were misclassified as high risk, despite an actual malignancy rate of only 2%. The RMI scoring system demonstrated a sensitivity of 0% and a specificity of 76% for this patient subset. Previous research has also indicated that the RMI performs inadequately in assessing borderline, early-stage, and non-epithelial tumors.

CT features of fibroma-thecoma tumors typically present as unilateral, well-defined, round, or oval solid masses (Figure 9) [41]. Differentiating these tumors from other benign ovarian lesions can be challenging. Smaller tumors generally exhibit homogeneous density, whereas larger tumors (greater than 60 mm) often present heterogeneous density due to cystic degeneration or necrosis. In a study, 73% of the tumors were solid, with 69% demonstrating heterogeneous density. Contrast enhancement in thecoma-fibroma tumors is usually minimal, showing no significant differences across CT phases, and consistently lower enhancement compared to normal myometrium on both CT and MRI [41, 42]. Most lesions display a plateau on the time-density curve after a mild initial enhancement. These findings suggest poor vascularity and align with prior CT and ultrasound studies indicating low or absent blood flow.

**Figure 10.**
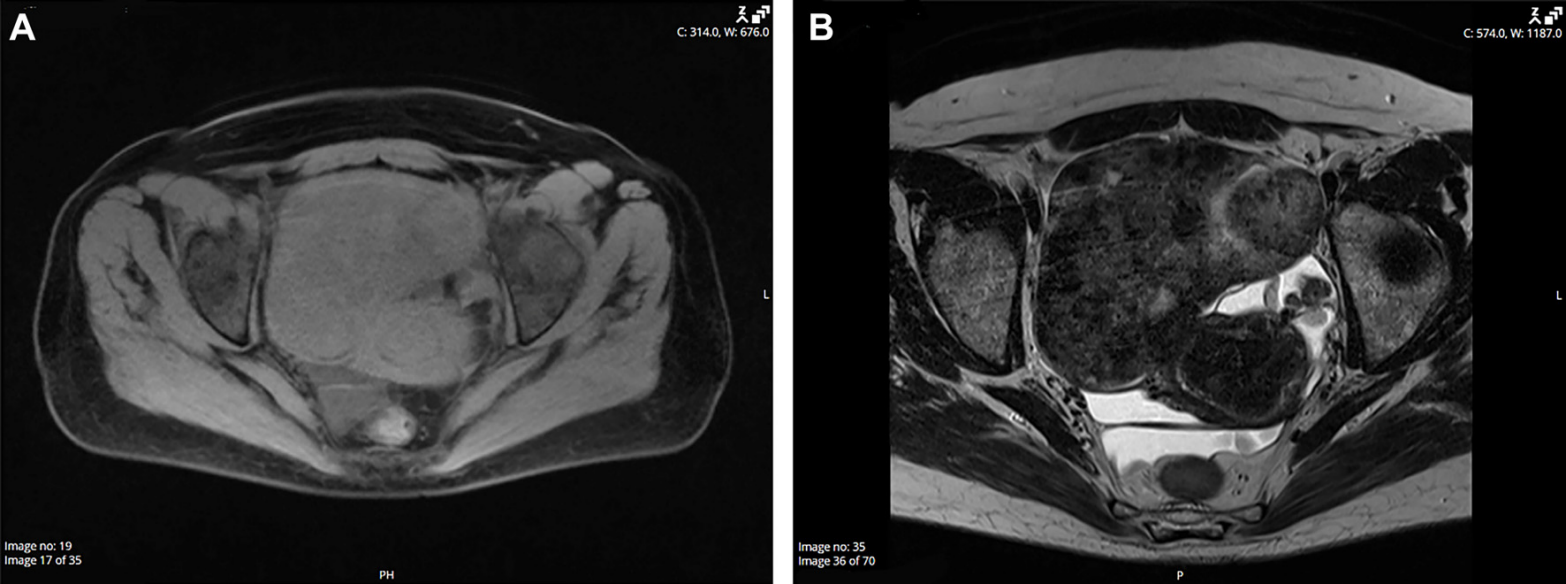
**MRI appearance of an ovarian fibroma.** Axial pelvic MRI shows a well-circumscribed, oval, lobulated right-adnexal mass contiguous with the uterus, measuring 92 × 107 × 130 mm. (A) T1-weighted fat-suppressed image: Predominantly isointense to myometrium; (B) T2-weighted image: Heterogeneously low signal with scattered hyperintense foci, in keeping with fibrous content. Mild–moderate heterogeneous post-contrast enhancement was present on delayed images (not shown). Histology confirmed an ovarian fibroma. MRI: Magnetic resonance imaging.

MRI, particularly T2-weighted sequences, provides superior soft-tissue contrast and is more effective in identifying the characteristic features of fibroma-thecomas, making it the preferred imaging modality for diagnosing these tumors [43]. In two separate studies, ovarian fibromas-thecomas consistently presented as well-defined masses, most commonly oval, round, or lobulated in shape, with sharp contours irrespective of size or form [14, 43]. On MRI, these tumors typically exhibited isointense to hypointense signals compared to the uterine muscle on both T1- and T2-weighted images (Figure 10). T1 signals were generally homogeneous, while T2 signals displayed greater heterogeneity, indicating internal structural variation. A thin, enhancing capsule was often discernible, particularly on T2-weighted sequences. Three distinct T2 signal patterns were identified: homogeneous low signal (indicative of fibrous content), isointense with scattered hyperintensities (suggesting edema or cellularity), and predominantly cystic lesions. Most tumors exhibited homogeneous post-contrast enhancement; however, some demonstrated mild or heterogeneous enhancement.

### Molecular studies

Trisomy 12 represents the most prevalent chromosomal abnormality identified in ovarian fibromas [44]. Chromosomal abnormalities are observed more frequently in pure fibromas (88%) compared to benign serous ovarian tumors (33%). This discrepancy implies that certain serous tumors may, in fact, be fibromas accompanied by incidental epithelial cysts. Although trisomy 12 is the sole chromosomal aberration identified more frequently in benign serous tumors (47%) than in fibromas (30%), fibromas demonstrate a higher incidence of copy number alterations, such as the gain of 9q (50%). This finding suggests a distinct underlying biology between these tumor types.

Molecular analysis has proven valuable in addressing complex differential diagnoses involving fibrothecomas and AGCTs [45]. The FOXL2 c.402C>G mutation was identified in 16 of 17 tumors (94.1%) ultimately diagnosed as AGCT, as well as in one case of gynandroblastoma. Five of these tumors had initially been classified morphologically as thecoma or fibrothecoma; however, the presence of the FOXL2 mutation prompted a revised diagnosis of AGCT. In contrast, no FOXL2 mutations were detected in tumors diagnosed as CFs, MACF s, or fibromas-thecomas. In these instances, the negative mutation status provided diagnostic validation for these entities, particularly when AGCT was a significant consideration. Among 13 additional tumors where AGCT was part of the provisional diagnosis, the absence of FOXL2 mutations facilitated alternative final diagnoses, including CF, unclassified sex cord–stromal tumor, juvenile granulosa cell tumor, or thecoma.

### Management

The treatment for these tumors is primarily surgical, utilizing either laparotomy or laparoscopy [15]. A study involving 97 patients indicates that the majority underwent minimally invasive laparoscopic surgery, which resulted in shorter operation times and no major complications [12]. Ovarian surgical procedures, including salpingo-oophorectomy, oophorectomy, and tumorectomy, were performed on most patients, with tumorectomy being the most prevalent choice, particularly among younger women seeking fertility preservation. In certain instances, hysterectomy in conjunction with ovarian surgery was necessary, often due to concomitant uterine conditions or concerns regarding malignancy, especially in postmenopausal patients. Notably, 90% of tumorectomies were conducted laparoscopically, predominantly in younger patients with a median age of 34, underscoring laparoscopy as a safe and effective option for fertility preservation.

Despite their generally benign nature, recurrences of fibroma-thecomas have been documented. One case recurred 10 years post-tumorectomy [46], while another patient experienced multiple recurrences over a nine-year period, including extragonadal lesions in the pelvic cavity. These findings suggest a potential for recurrence, even in histologically benign tumors [47].

Managing rare benign tumors that mimic malignancies presents a significant clinical challenge, as diagnostic uncertainty may result in overtreatment. Similar concerns regarding recurrence risk and surgical strategy have been documented in other misleading ovarian conditions [48], such as struma ovarii. This underscores the necessity for meticulous preoperative planning and personalized surgical decision-making.

### Differential diagnosis

#### Fibroma-thecoma tumors

Accurate preoperative diagnosis of ovarian thecoma tumors remains challenging due to their overlapping imaging characteristics with other pelvic masses. In one study, ultrasound-based preoperative diagnoses accurately identified ovarian thecoma tumors in only 72.13% of cases. The remaining cases were misclassified as subserous myomas (19.67%), ovarian malignancies (1.64%), endometriomas (3.28%), complex cysts (1.64%), or cystadenomas (1.64%) [25]. Similarly, another study revealed that numerous patients with ovarian masses were initially misdiagnosed with uterine myomas, resulting in unexpected intraoperative findings of fibromas [12]. This preoperative misdiagnosis led to unnecessary hysterectomies for affected patients.

The primary differential diagnoses for ovarian fibromas-thecomas include Brenner tumors, granulosa cell tumors, dysgerminomas, and pedunculated subserosal or broad ligament leiomyomas [14].

The presence of nuclear atypia, necrosis, and hemorrhage should raise suspicion for fibrosarcoma; however, it is crucial to differentiate this condition from the mitotically active variant of CF, as previously discussed [9].

AGCTs can pose significant diagnostic challenges, particularly when they exhibit extensive thecoma-like areas and share features with fibromas and CFs [49]. A subset of AGCTs, commonly found in postmenopausal women and often discovered incidentally, may present with a solid, yellow nodular architecture and cytological features that closely resemble thecomas. These features include cells with abundant pale cytoplasm and occasional sclerosis or calcification. Notably, classic granulosa cell characteristics, such as Call-Exner bodies or grooved nuclei, may be absent or only present in a focal manner, complicating morphological distinctions.

Differentiating between thecoma and granulosa cell tumor can be facilitated by reticulin staining [49, 50]. In thecomas, reticulin fibers typically envelop individual tumor cells, creating a dense pericellular network. Conversely, granulosa cell tumors often demonstrate a reduction or absence of reticulin around individual cells, with staining instead outlining nests or larger clusters, reflecting their epithelial differentiation. Consequently, a reticulin-poor area resembling thecoma may indicate granulosa cell neoplasia. AGCTs are also associated with mutations in the *FOXL2* gene, as previously discussed [45]. However, it is crucial to recognize that FOXL2 immunohistochemistry is not mutation-specific. One study found that while 98% of sex-cord stromal tumors with FOXL2 mutations expressed FOXL2, approximately one-third of FOXL2 wild-type tumors also exhibited expression, thereby limiting its specificity [21]. Furthermore, AGCTs characteristically demonstrate immunoreactivity for markers such as S100, CD99, CD56, and SMA, a profile typically absent in fibromas and thecomas.

Brenner tumors and dysgerminomas are solid ovarian masses that typically exhibit low signal intensity on T2-weighted imaging but are considered rare [14]. Leiomyomas may mimic fibromas or thecomas, particularly when degenerated, but they usually display early, pronounced enhancement.

Several entities warrant consideration in the differential diagnosis of thecoma, particularly among other stromal tumors. Sclerosing stromal tumors may mimic thecoma due to their lobulated growth; however, they typically exhibit a mixture of cell types and prominent vascularity, which are not characteristic of thecoma [50]. Microcystic stromal tumors often demonstrate cystic changes, occur in younger patients, and are positive for CD10. These tumors can pose diagnostic challenges, as they may contain hyaline plaques typically associated with thecomas. Luteinized thecomas with sclerosing peritonitis, despite their name, are distinct from conventional thecomas and rarely lead to diagnostic confusion. These rare tumors are characterized by cortical spindle cell proliferation with scattered luteinized cells [51]. Spindle cells often lack typical sex cord markers, while luteinized cells express α-inhibin and calretinin. Peritoneal lesions exhibit bland myofibroblastic proliferation without atypia, and the presence of ascites is common, albeit non-malignant. CFs and MACFs can resemble other ovarian spindle cell tumors, including cellular leiomyomas, endometrioid stromal sarcomas (ESSs), GISTs, and non-neoplastic stromal hyperplasia [9]. Leiomyomas display eosinophilic cytoplasm, blunt-ended nuclei, and positivity for smooth muscle markers. While ESSs may mimic CFs/MACFs, they are often associated with endometriosis or uterine tumors and are CD10 positive. GISTs can present similarly, sometimes preceding or following the identification of the primary tumor, and are characterized by c-kit positivity. Stromal hyperplasia, typically bilateral, shows less cellular atypia and minimal collagen deposition. Immunohistochemistry and the clinical context are crucial for accurate diagnosis.

#### Extraovarian tumors

Tumors classified as originating from the broad ligament must be located within or on the ligament itself and must be entirely separate from the uterus and ovaries, with no anatomical connection to these structures [52]. Consequently, the initial step in differential diagnosis involves ruling out uterine, ovarian, or tubal origins by clearly identifying the uterus and ovaries as distinct entities and evaluating imaging for any signs of continuity. Transvaginal ultrasound, typically the first imaging modality employed, can indicate a broad ligament origin when it shows a clear separation of the mass from the adjacent reproductive organs. However, magnetic resonance imaging (MRI) provides superior multiplanar capabilities and greater accuracy in confirming the tumor’s origin and assessing its relationship to surrounding structures. Additionally, MRI offers critical information regarding tumor size, lymphadenopathy, and potential metastases, establishing it as the preferred method for preoperative evaluation.

Leiomyoma is the most prevalent primary tumor of the broad ligament, although it remains rare, accounting for less than 1% of cases [52]. This tumor originates from smooth muscle fibers within the connective tissue of the ligament and histologically resembles uterine leiomyomas. Differentiating true broad ligament leiomyomas from uterine tumors that extend into the ligament can be challenging, particularly in large masses that distort pelvic anatomy or compress the ureters. Additionally, rare mimics include GISTs, which are positive for c-Kit and DOG-1, and perivascular epithelioid cell tumors, which are positive for HMB-45. These tumors may histologically resemble leiomyomas but differ in their immunohistochemical profiles [53].

A diagnosis based solely on histopathological evaluation is inadequate due to the overlapping characteristics with leiomyomas, GISTs, and fibromatosis, thereby underscoring the necessity of immunohistochemical analysis. The expression of PRs confirms the ovarian origin of the tumor, while the absence of CD34, c-Kit, and desmin effectively excludes leiomyomas and GISTs [54]. In desmoid-type fibromatosis, tumor cells demonstrate nuclear beta-catenin expression, a feature that is notably absent in fibromas.

Multiple theories have been proposed regarding the origin of extraovarian fibrothecomas. These theories suggest development from accessory ovaries (located adjacent to the normal ovary), supernumerary ovaries (distinct third ovaries), or Müllerian remnants or mesothelial tissue [31]. Despite their extraovarian location, fibroma-thecomas exhibit histological characteristics identical to those of their ovarian counterparts. Tumors predominantly composed of sex cord elements have been discussed in [3]. Histological and immunohistochemical analyses confirm that these tumors correspond to ovarian fibrothecomas.

### Strenghts and limitations

This study presents one of the few documented cases of extraovarian fibrothecomas, accompanied by a detailed clinicopathological correlation, extensive immunohistochemical analysis, and imaging review, thereby providing valuable insights into this rare entity. The comprehensive differential diagnosis and comparison with existing literature enhance its diagnostic relevance. However, the study’s case report design limits its generalizability. The small sample size and lack of long-term follow-up data hinder broader conclusions regarding prognosis or recurrence. Furthermore, the absence of molecular testing restricts characterization at the molecular level, limiting deeper insights into pathogenesis and potential diagnostic markers. Additionally, due to the retrospective nature of this report, certain clinical and laboratory data may be incomplete.

## Conclusion

These cases represent rare instances of extraovarian fibrothecomas, a benign and surgically curable tumor that is typically found within the ovary. Since its initial recognition, only 15 cases have been reported at extraovarian sites. Accurate diagnosis is critical, as these tumors can closely mimic malignancy due to their association with ascites, elevated CA-125 levels, and, in rare cases, pleural effusion, resembling Meigs syndrome, a condition typically linked to ovarian fibromas. The etiology of extraovarian fibrothecomas is still unclear, so it is of particular importance to present each diagnosed case with a detailed histomorphology and differential diagnosis, as it is in our report. The pathologist should be aware of the association with elevated level of CA-125, one of the controversial aspects of extraovarian fibrothecoma, to avoid misinterpretation as malignancy. Besides the increased level of CA-125, the diagnosis of the second case was complicated with ascites and pleural effusion, resembling Meigs syndrome, which is mostly associated with ovarian fibromas. However, as the effusion did not resolve spontaneously and required drainage, thus does not fulfill the classic criteria for Meigs syndrome. We undertook a very detailed microscopic analysis, wide specific immunostaining, and a review of the literature, in order to establish the right diagnosis with good prognosis, and to share the experience of this still debatable entity.

## Data Availability

Data are available from the corresponding author upon reasonable request.
